# BRG1 Deficiency Promotes Cardiomyocyte Inflammation and Apoptosis by Activating the cGAS-STING Signaling in Diabetic Cardiomyopathy

**DOI:** 10.1007/s10753-024-02058-7

**Published:** 2024-06-13

**Authors:** Ziying Chen, Xiangmao Lai, Jingxuan Li, Xun Yuan, Yilang Li, Xiaojing Zhang, Zhanfang Kang, Zizhang Ouyang, Jianwen Zeng, Ning Hou, Xiaoping Liu

**Affiliations:** 1 Department of Pharmacy, Affiliated Qingyuan Hospital, Guangzhou Medical University, Qingyuan People’s Hospital, Qingyuan 511518, China; 2 Guangdong Key Laboratory of Molecular Target & Clinical Pharmacology, the State Key Laboratory of Respiratory Disease, School of Pharmaceutical Sciences and the Fifth Affiliated Hospital, Guangzhou Medical University, Guangzhou, 511436 China; 3 Department of Urology, Affiliated Qingyuan Hospital, Guangzhou Medical University, Qingyuan People’s Hospital, Qingyuan, 511518 China; 4 Guangdong Engineering Technology Research Center of Urinary Continence and Reproductive Medicine, Guangzhou Medical University, Qingyuan, 511518 China; 5 Department of Basic Medical Research, Affiliated Qingyuan Hospital, Guangzhou Medical University, Qingyuan People’s Hospital, Qingyuan, 511518 China

**Keywords:** brahma-related gene 1, cardiomyocyte apoptosis, cGAS, diabetic cardiomyopathy, STING

## Abstract

Brahma-related gene 1 (BRG1) has been implicated in the repair of DNA double-strand breaks (DSBs). Downregulation of BRG1 impairs DSBs repair leading to accumulation of double-stranded DNA (dsDNA). Currently, the role of BRG1 in diabetic cardiomyopathy (DCM) has not been clarified. In this study, we aimed to explore the function and molecular by which BRG1 regulates DCM using mice and cell models. We found that BRG1 was downregulated in the cardiac tissues of DCM mice and in cardiomyocytes cultured with high glucose and palmitic acid (HG/PA), which was accompanied by accumulation of dsDNA and activation of the cyclic GMP-AMP synthase (cGAS)–stimulator of interferon genes (STING) signaling pathway. shRNA-mediated *Brg1* knockdown aggravated DCM mice cardiac functions, enhanced dsDNA accumulation, cGAS-STING signaling activation, which induced inflammation and apoptosis. In addition, the results were further verified in HG/PA-treated primary neonatal rat cardiomyocytes (NRCMs). Overexpression of BRG1 in NRCMs yielded opposite results. Furthermore, a selective cGAS inhibitor RU.521 or STING inhibitor C-176 partially reversed the BRG1 knockdown-induced inflammation and apoptosis *in vitro*. In conclusion, our results demonstrate that BRG1 is downregulated during DCM *in vivo* and *in vitro*, resulting in cardiomyocyte inflammation and apoptosis due to dsDNA accumulation and cGAS-STING signaling activation. Therefore, targeting the BRG1-cGAS-STING pathway may represent a novel therapeutic strategy for improving cardiac function of patients with DCM.

**Supplementary Information:**

The online version contains supplementary material available at 10.1007/s10753-024-02058-7.

## Introduction

Diabetes, a non-communicable metabolic disease, is increasingly becoming a major pandemic worldwide [1]. Although great progress has been made in the establishment of diabetes treatment, the available treatments have several chronic complications, especially cardiovascular diseases [2]. DCM is the main cause of death in diabetic patients, and is characterized by myocardial hypertrophy, cardiac fibrosis, and heart failure [3]. The pathophysiology of DCM is complex with multiple pathophysiological mechanisms associated with its, including mitochondrial dysfunction, myocardial inflammation, and apoptosis [4, 5]. Uncontrolled myocardial inflammation and apoptosis are key processes that cause cardiac dysfunction in DCM [6]. Therefore, expanding our understanding of the complexity of DCM pathophysiology, particularly the identification of novel genes and regulatory pathways, will help to identify treatments for DCM-induced cardiomyocyte inflammation and apoptosis.

BRG1, also known as *SMARCA4*, encodes a component of the switch/sucrose nonfermentable (SWI/SNF) complex, which regulates the ATPase and helicase activities. Recent studies have demonstrated that BRG1 downregulation increased the transcription of proinflammatory genes and induced apoptosis [7, 8]. A previous study demonstrated that BRG1 participates in cardiac growth and differentiation processes [9]. In our earlier study, we found that BRG1 was upregulated during acute myocardial infarction, and BRG1 overexpression alleviated cardiomyocyte oxidative damage and increased cardiomyocyte viability [10]. In addition, BRG1 upregulation was found to ameliorate diabetic cardiomyopathy-induced diastolic dysfunction [11]. However, whether BRG1 is involved in the development of DCM remains to be determined.

Studies have implicated BRG1 in the repair of DNA double-strand breaks (DSBs), loss of BRG1 impairs DSBs repair resulting in the accumulation of cytoplasmic dsDNA [12, 13]. cGAS, a dsDNA sensor, uses cytosolic DNA to generate cyclic GMP–AMP, which binds to its receptor STING. Subsequently, STING activates the transcription factor nuclear factor-kappa B (NF-κB) and interferon regulatory factor 3 (IRF3) *via* the TANK binding kinase 1 (TBK1) [14, 15]. Recent studies have confirmed that cGAS–STING also contributes to DCM by sensing mitochondrial damage-released DNA [16, 17]. Therefore, studies are needed to explore the relationship between BRG1 expression and the cGAS–STING during the pathogenesis of DCM.

In this study, we utilize an HFD and streptozotocin (STZ)-induced DCM mouse model and HG/PA-treated cardiomyocytes injury model to investigate the relationship between BRG1 expression and cGAS-STING activation in DCM. BRG1 was knocked down in DCM mice and HG/PA-treated cardiomyocytes to deeply explore the mechanism by which BRG1 regulates DCM. These results show that BRG1 has promising therapeutic potential for application in DCM.

## Materials and Methods

### Vector and Adeno-associated Virus; Lentivirus Construction and Adenovirus

The adeno-associated virus (AAV) vector carrying cardiac troponin T (CTNT) promoter, green fluorescent protein (GFP) and *Brg1* shRNA were purchased from Hanbio Biotechnology Co. Ltd (Shanghai, China). The cardiomyocyte-specific CTNT promoter promotes the expression of *Brg1* shRNA in cardiomyocytes. The lentiviral virus vector carrying GFP and *Brg1* shRNA was constructed by Cyagen Biosciences (Guangzhou, China). The *Brg1* shRNA sequence was 5′-GCTGCCAAATACAAACTCAATCTCGAGATTGAGTTTGTATTTGGCAGC-3′. The adenovirus carrying GFP and *Brg1*-adenovirus were constructed by the WZ Biosciences Inc. (Shandong, China). The titers of the AAV stock, lentivirus stock and adenovirus stock were 1.68 × 10^12^ vg/ml, 3.98 × 10^9^ TU/ml and 1 × 10^10^ pfu/ml, respectively.

### Animal Experiments

All animal procedures were approved by the Institutional Animal Care and Use Committee of Affiliated Qingyuan Hospital, Guangzhou Medical University (Guangdong, China). 3–4-weeks old male C57BL/6J mice were purchased from Vital River Laboratory Animal Technology Co., Ltd (Guangdong) and housed under specific pathogen-free condition with a 12 h light/dark cycle, and 25 ± 1 °C, 60 ± 5% humidity. The mice were randomly divided into six groups: control (CON), control with AAV 9-scramble (AAV-scramble), control with myocardium-specific knockdown of *Brg1* shRNA (AAV-*Brg1* shRNA), DCM, DCM with AAV-scramble, and DCM with AAV-*Brg1* shRNA. They were then intravenously injected with AAV-*Brg1* shRNA into the tail vein to establish a model of myocardium-specific knockdown of *Brg1*. The diabetic mice were subsequently intraperitoneally injected with streptozotocin (STZ; 85 mg/kg, Sigma Aldrich, USA) twice and fed on HFD. Mice in the control group were given a normal diet and injected with the same volume of citrate sodium buffer. After injection, a contour glucose meter (Johnson & Johnson, USA) was used to measure blood glucose levels at 3, 5, and 7 days, and postprandial blood glucose ≥ 16.7 mmol/L indicated diabetes. After 16 weeks of treatment, cardiac function was evaluated using the Vevo2100 system.

### Echocardiography

Mice electrocardiography was performed using a transthoracic echocardiography (Vevo2100; Visual Sonics, Canada). Briefly, mice were anesthetized with 2% isoflurane, and cardiac function parameters were measured, including the E/A ratio, left ventricular ejection fraction (LVEF), left ventricular fractional shortening (LVFS), and other left ventricular (LV) parameters.

### Isolation of NRCMs and Treatment

The NRCMs were isolated from 1–2 days Sprague Dawley rats as previously described [18]. Cultured cardiomyocytes were transfected with Ad-Mock, Ad-*Brg1* WT [the multiplicity of infection (MOI) = 5]; Lenti-scramble, or Lenti-*Brg1* shRNA (the MOI = 25) for 48 h, or pretreated with 10 µmol RU.521 (Cat. #HY-114180; New Jersey city, USA) or C-176 (Cat. # HY-112906; New Jersey city, USA) for 24 h and then incubated with 33 mM glucose and 300 μM palmitic acid for 48 h. The NRCMs were subsequently harvested for analysis.

### Histology and Immunofluorescent Staining

Fresh mice hearts were fixed in 10% formalin solution and embedded in paraffin. The heart was sectioned into 4 μm thick sections and stained with Masson's and hematoxylin-eosin (HE) staining.

*In vitro,* cardiomyocytes were fixed in 4% paraformaldehyde. The membranes were permeabilized with 1% triton and incubated with 100 nM glycine. Subsequently, they were blocked with 10% goat serum for 1 h followed by incubation with primary antibody overnight at 4 °C. On the following day, cardiomyocytes or tissue sections were incubated with dylight 561-coupled anti-mouse IgG and dylight 488/647-coupled anti-rabbit IgG. Finally, the nuclei were stained with DAPI at a concentration of 0.5 μg/ml. The sections were imaged using a confocal microscope (LSM900). The antibody information is presented in Table S1.

### Western Blotting (WB) Assessment

Cells or tissues were lysed to extract proteins, which was then separated by 10–15% sodium dodecyl-sulfate polyacrylamide gel electrophoresis gel (SDS-PAGE) and transferred to polyvinylidene difluoride (PVDF) membrane. The PVDF membranes were blocked with 5% skimmed milk and incubated with primary antibodies overnight at 4 °C. The membranes were then incubated with a secondary antibody. Finally, the protein bands were analyzed using the gel imaging system (Biorad ChemiDoc). The details of antibodies used are presented in Table S1.

### Quantitative Real-time PCR (qRT-PCR)

The total RNA was extracted from treated cardiomyocytes or cardiac tissues using TRIzol reagent (Invitrogen). The RNA was reverse transcribed into complementary DNA (cDNA) using the Takara PrimeScript™ RT Master Mix (Takara Bio Inc., Japan). The cDNA was subjected to qPCR amplification on the CFX Connect™ Real-Time system (Biorad). The primers used in this experiment were designed by the NCBI Primer-BLAST and are listed in Table S2.

### TdT-mediated dUTP Nick End Labeling (TUNEL) Staining

The apoptosis rate of cardiomyocytes was determined by TUNEL staining. Briefly, mice myocardial tissues were excised, fixed, paraffinized and then sectioned. *In vitro,* cardiomyocytes were fixed by 4% paraformaldehyde solution. The membranes were permeabilized with 1% triton and incubated with 100 nM glycine. The incubation buffer was the prepared following instructions on the TUNEL kit (Cat. 12156792910 Roche Diagnostics, Germany), and was applied in a constant temperature incubator at 37 °C in darkness. The nuclei were stained with DAPI at a concentration of 0.5 μg/ml. Confocal microscope (LSM900) was employed to examine and capture images of the slices for analysis.

### Statistical Analysis

Statistical analysis was performed by GraphPad Prism 8.0 (San Diego, CA, USA), and the data were expressed as the mean ± standard error. Groups were compared with paired t-tests or one-way ANOVAs with *post-hoc* Tukey tests. *P* < 0.05 was considered statistically significant.

## Results

### BRG1 is Downregulated in the Heart of DCM Mice

**Fig. 1:**
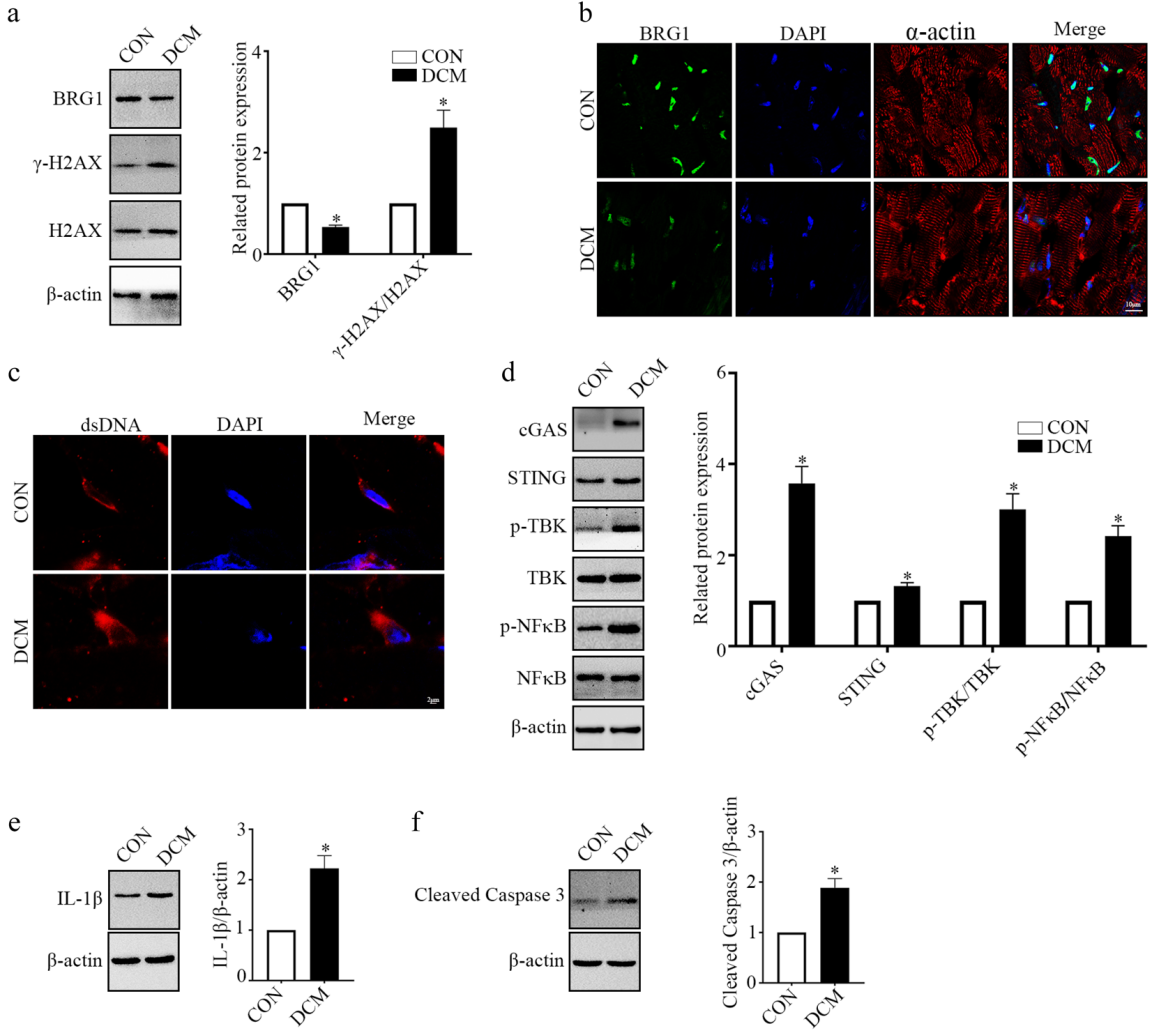
BRG1 is downregulated in the heart of DCM mice. **a** Protein levels of BRG1 and γ-H2AX were assayed using Western blot in mice cardiac tissues. **b** Representative immunofluorescence images of BRG1 in cardiac tissues. **c** The accumulation of dsDNA was evaluated using immunofluorescent staining in mice cardiac tissues. **d** Protein levels of cGAS-STING signaling-related genes were assayed using Western blot in mice cardiac tissues. **e** Level of IL-1β protein was assayed using Western blot in mice cardiac tissues. **f** Level of cleaved caspase-3 was assayed using Western blot in mice cardiac tissues. * indicates *P* < 0.05 vs. the CON group.

In this study, we established a DCM mouse model by feeding a HFD and administering STZ. We initially assessed the BRG1 protein levels in the cardiac tissues of DCM mice. Western blot analysis revealed that BRG1 protein level was decreased in the cardiac tissues of DCM mice compared to normal mice (Fig. 1a). Immunofluorescence staining further confirmed the localization and decreased expression of BRG1 in the hearts of DCM mice (Fig. 1b). Considering that BRG1 participates in the repair of DSBs, we detected the accumulation of H2AX phosphorylation (γ-H2AX, a surrogate marker for DSBs) and dsDNA in the mice cardiac tissues. It was observed that the γ-H2AX protein level was higher in cardiac tissues from the DCM mice compared with the normal mice (Fig. 1a). In addition, the cytoplasmic dsDNA content was increased in the heart tissues (Fig. 1c). Consistent with these results, the protein levels of cGAS, STING, p-TBK, and p-NF-κB were significantly upregulated in cardiac tissues from the DCM mice (Fig. 1d). Furthermore, analysis of the expression level of inflammation and apoptosis markers in the pathogenesis of DCM revealed that the concentration of IL-1β and cleaved caspase-3 were upregulated in the cardiac tissues from DCM mice (Fig. 1e and f). These results suggested that BRG1 was downregulated in the heart tissues of DCM model, which may result in cGAS-STING signaling activation, inflammation and apoptosis.

### BRG1 Expression is Downregulated in HG/PA-treated Cardiomyocytes

**Fig. 2:**
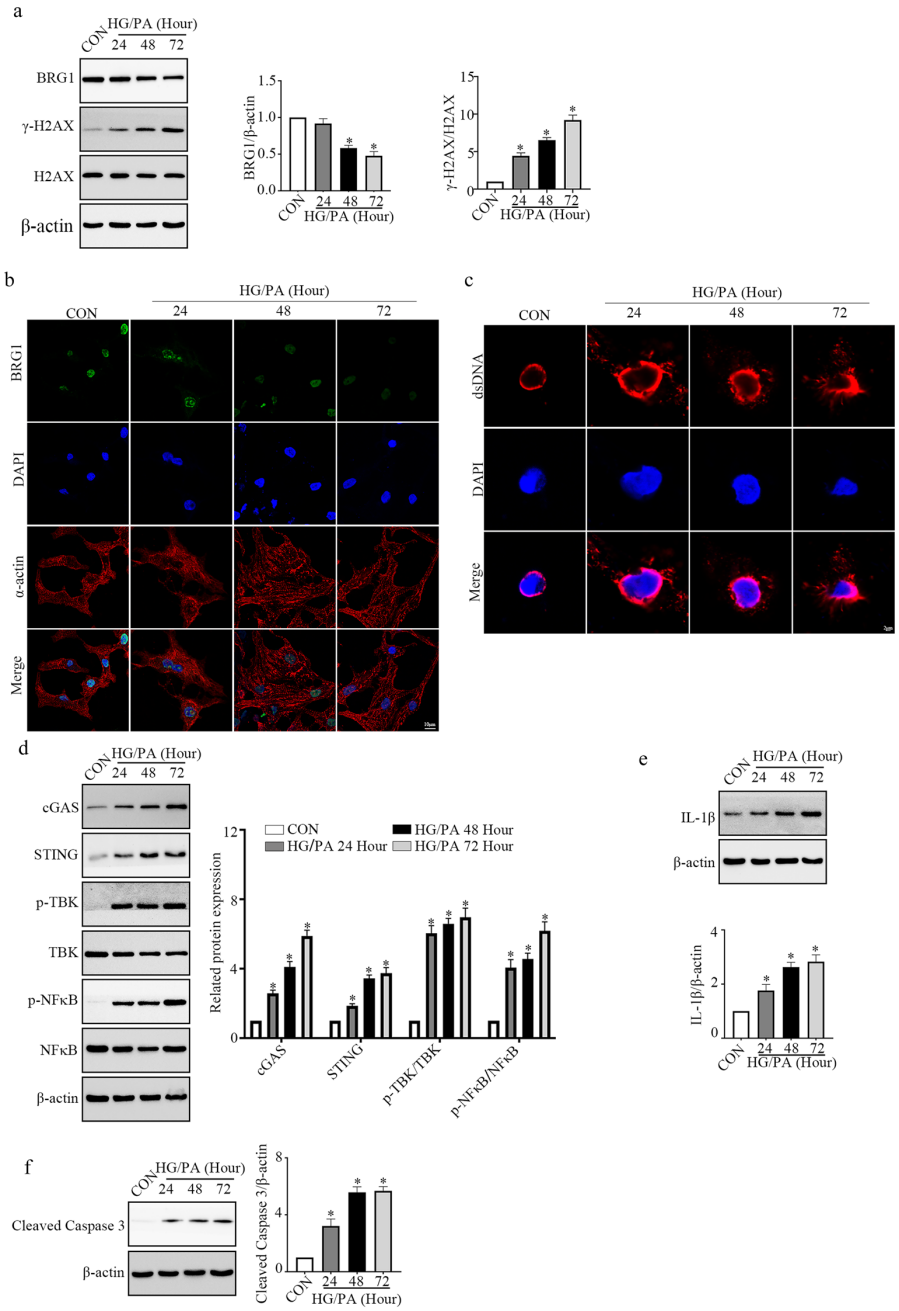
BRG1 expression is downregulated in HG/PA-treated cardiomyocytes. **a **Protein levels of BRG1 and γ-H2AX were assayed using Western blot. **b** Representative immunofluorescence images of BRG1 in cardiomyocytes. **c** The accumulation of dsDNA was evaluated using fluorescent staining. **d** Protein levels of cGAS-STING signaling-related genes were assayed using Western blot. **e** Level of IL-1β protein was assayed using Western blot. **f** Level of cleaved caspase-3 protein was assayed using Western blot. * indicates *P* < 0.05 vs. the CON group.

Primary cardiomyocytes were incubated with HG/PA to mimic the hyperglycemia and hyperlipemia *in vitro*. Subsequently, the expression levels of BRG1 and γ-H2AX in the HG/PA-treated cardiomyocytes were measured. Compared with the control group, BRG1 expression was gradually decreased in a time-dependent manner following HG/PA stimulation, whereas the expression of γ-H2AX was gradually increased (Fig. 2a). The immunofluorescence results confirmed the localization and downregulation of BRG1 expression in HG/PA-cultured cardiomyocytes (Fig. 2b). Furthermore, we evaluated the cytoplasmic dsDNA content and the activity of the cGAS–STING in the HG/PA-treated cardiomyocytes. Data shown in Fig. 2c suggested that the cytoplasmic dsDNA content was increased following HG/PA treatment. In addition, the protein levels of cGAS, STING, p-TBK, and p-NF-κB were significantly upregulated in HG/PA-treated cardiomyocytes (Fig. 2d). Consistent with this, the expression levels of IL-1β and cleaved caspase-3 were upregulated in HG/PA-treated cardiomyocytes in a time-dependent manner (Fig. 2e and f). Altogether, these results indicated that BRG1, dsDNA, and the cGAS–STING co-regulate inflammation and apoptosis in HG/PA-treated cardiomyocytes.

### BRG1 Deficiency Aggravated Cardiac Dysfunction *In Vivo*

**Fig. 3:**
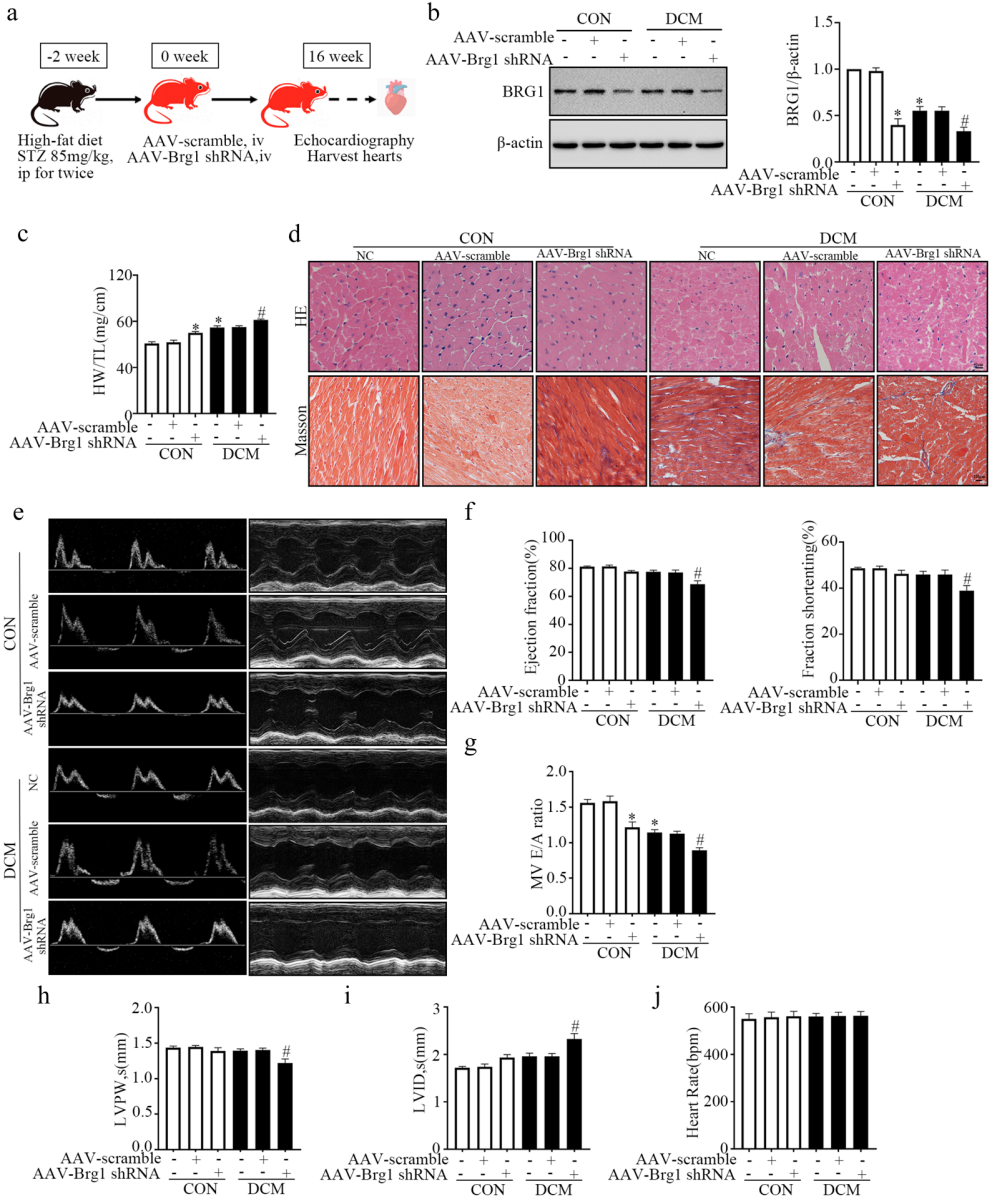
BRG1 deficiency aggravates experimental mouse cardiac dysfunction *in vivo*. **a** Schematic illustration for mouse experimental design. **b** Level of BRG1 protein was assayed using Western blot in mice cardiac tissues. **c** Calculation and analysis of the heart weight/tibial length (HW/TL) ratio. **d** The HE staining and Masson's staining of the mouse heart tissues. **e** Representative M-mode echocardiographic imaging. **f** Calculation of ejection fraction and fractional shortening of heart. **g** E/A ratio. **h** left ventricular posterior wall (LVPW) in end-systole. **i** left ventricular internal diameter (LVID) in end-systole. **j** Heart rate. * indicates *P* < 0.05 vs. the CON group. # indicates* P* < 0.05 vs. the DCM group.

To explore the function of BRG1 in DCM pathogenesis, we constructed a DCM mouse model with myocardium-specific knockdown of BRG1 *via* injection of AAV-*Brg1* shRNA. The experimental design of this process is shown in Fig. 3a. The GFP-tagged virus was used as a scrambled control. The *in vivo* imaging and GFP immunofluorescence results indicated that AVV targeted and was enriched in myocardial tissue (Supplementary Fig. 1A and B). In addition, the WB results confirmed that the protein level of BRG1 in heart tissue was effectively knocked down following AAV-*Brg1* shRNA injection (Fig. 3b). The heart weight/tibial length (HW/TL) ratio (Fig. 3c) and the sectional area of myocardial cross (Fig. 3d) were increased after AAV-*Brg1* shRNA injection. Analysis of the echocardiography results revealed significant reduction in the E/A ratio in the model group. Mice injected with AAV-*Brg1* shRNA exhibited significantly decreased in the E/A ratio compared to the control group (Fig. 3g).Compared to the DCM group, mice injected with AAV-*Brg1* shRNA exhibited significantly decreased echocardiographic parameters of LVEF, LVFS, E/A ratio, and LVPW in end-systole, while LVID in end-systole was significantly increased (Fig. 3e-i). In addition, the heart rates were comparable among the study groups (Fig. 3j). These results indicated that BRG1 contributed to the pathological process of DCM *in vivo*.

### BRG1 Deficiency Increased Cytoplasmic dsDNA Accumulation and Activated cGAS-STING Signaling

**Fig. 4:**
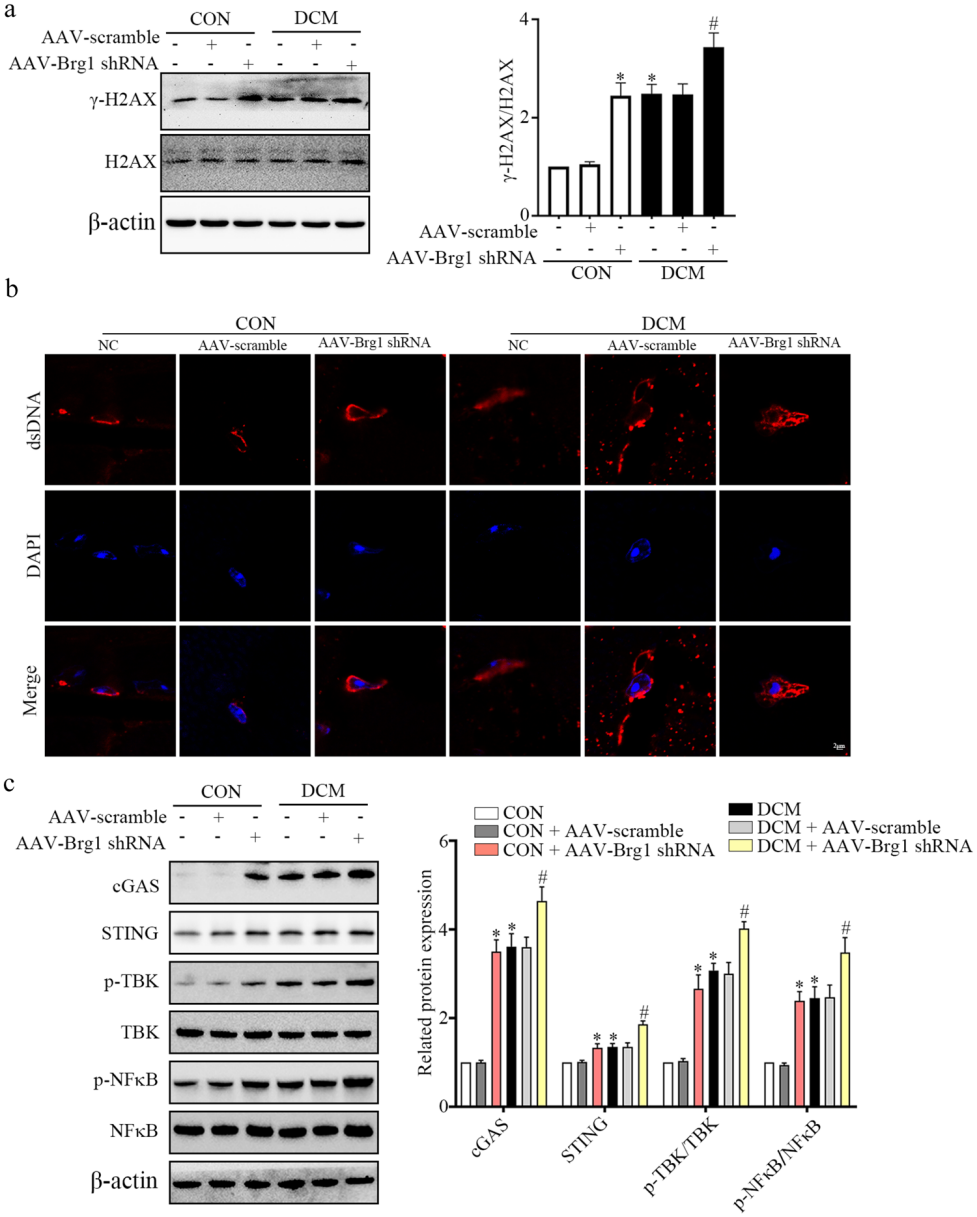
BRG1 deficiency increased cytoplasmic dsDNA accumulation and activated cGAS-STING signaling in mice cardiac tissues. **a** Level of γ-H2AX protein was assayed using Western blot in mice cardiac tissues. **b** The accumulation of dsDNA was evaluated using fluorescent staining. **c** Protein levels of cGAS-STING signaling-related genes were assayed using Western blot in mice cardiac tissues. * indicates *P* < 0.05 vs. the CON group, # indicates* P* < 0.05 vs. the DCM group.

To explore the role of BRG1 in the pathological mechanism of DCM, we assessed γ-H2AX expression, cytoplasmic dsDNA content, and activation of the cGAS-STING in DCM mice following cardiomyocyte-specific BRG1 knockdown. Our results revealed a significant increase in the protein level of γ-H2AX following AAV-*Brg1* shRNA injection (Fig. 4a). Consistent with this finding, the cytoplasmic dsDNA content was also increased after AAV-*Brg1* shRNA transfection (Fig. 4b). Furthermore, AAV-*Brg1* shRNA significantly upregulated the protein levels of cGAS, STING, p-TBK, and p-NF-κB in both control and DCM mouse cardiac tissues (Fig. 4c). Collectively, these results suggested that BRG1 knockdown increased the cytoplasmic dsDNA content and activated the cGAS-STING.

### BRG1 Deficiency Induced Inflammation and Apoptosis in Mouse Cardiac Tissues

**Fig. 5:**
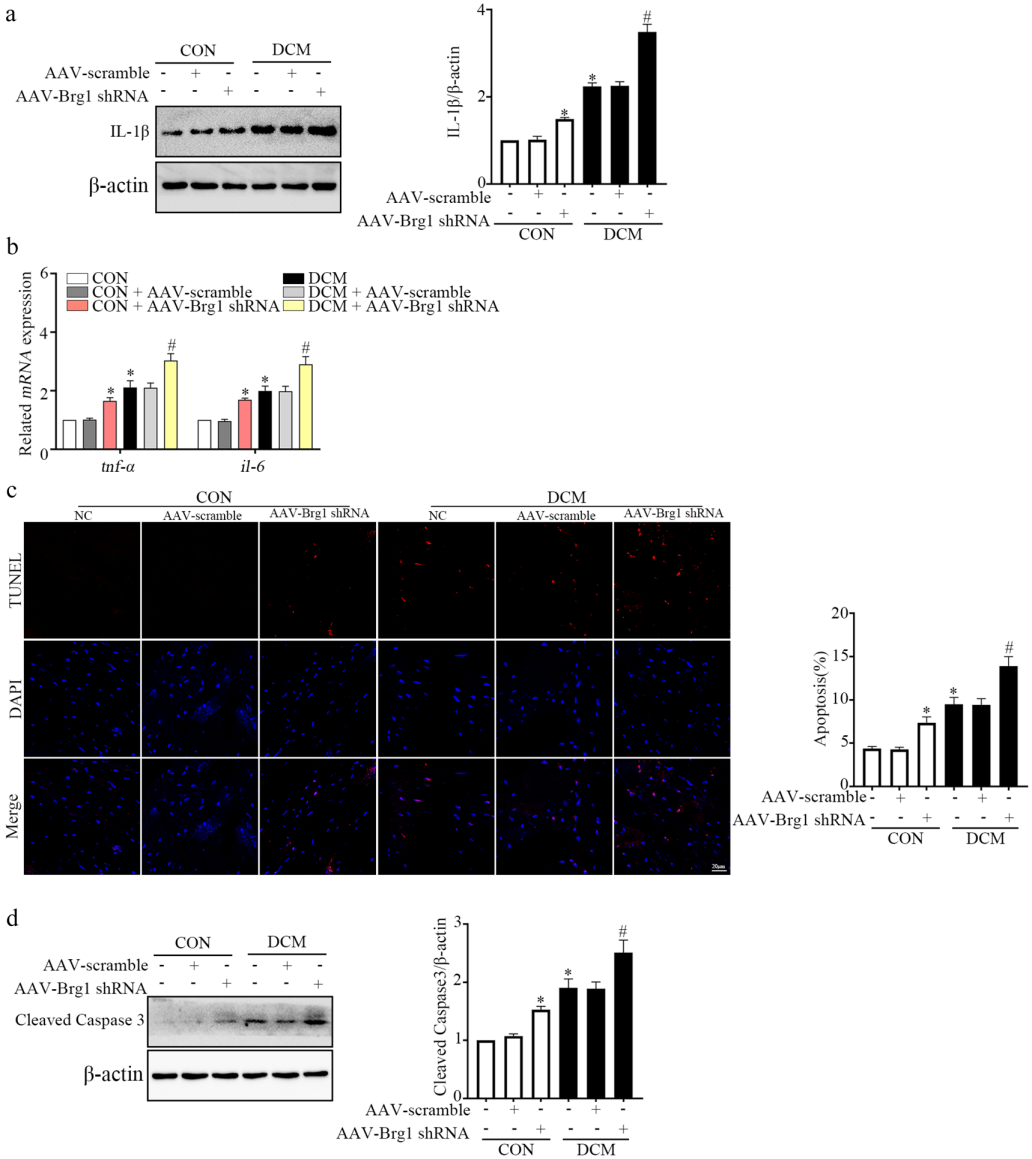
BRG1 deficiency induced inflammation and apoptosis in mouse cardiac tissues. **a** Level of IL-1β was assayed using Western blot in mice cardiac tissues. **b** *mRNA* levels of *tnf-α* and *il-6* were evaluated using qRT-PCR. **c** The apoptosis was detected by the TUNEL assay in mice cardiac tissues. **d** Level of cleaved caspase-3 was assayed using Western blot. * indicates *P* < 0.05 vs. the CON group, # indicates* P* < 0.05 vs. the DCM group.

Since dsDNA and cGAS-STING have close relationship with inflammation and apoptosis, we detected the concentration of markers in the cardiac tissues of AAV-*Brg1* shRNA transfected mice. The WB results indicated that the IL-1β protein level was significantly upregulated in the AAV-*Brg1* shRNA transfected group (Fig. 5a). Levels of *tnf-α* and *il-6* mRNA also exhibited similar trends (Fig. 5b). TUNEL straining results confirmed that knockdown of BRG1 increased the percentage of apoptotic cardiomyocytes (Fig. 5c). In addition, cleaved caspase-3 was upregulated in cardiac tissues of AAV-*Brg1* shRNA transfected mice (Fig. 5d). These findings demonstrated that knockdown of BRG1 induced inflammation and apoptosis in mice cardiac tissues.

### BRG1 Overexpression Inhibited HG/PA-induced Cytoplasmic dsDNA Accumulation and cGAS-STING Signaling Activation *In Vitro*

**Fig. 6:**
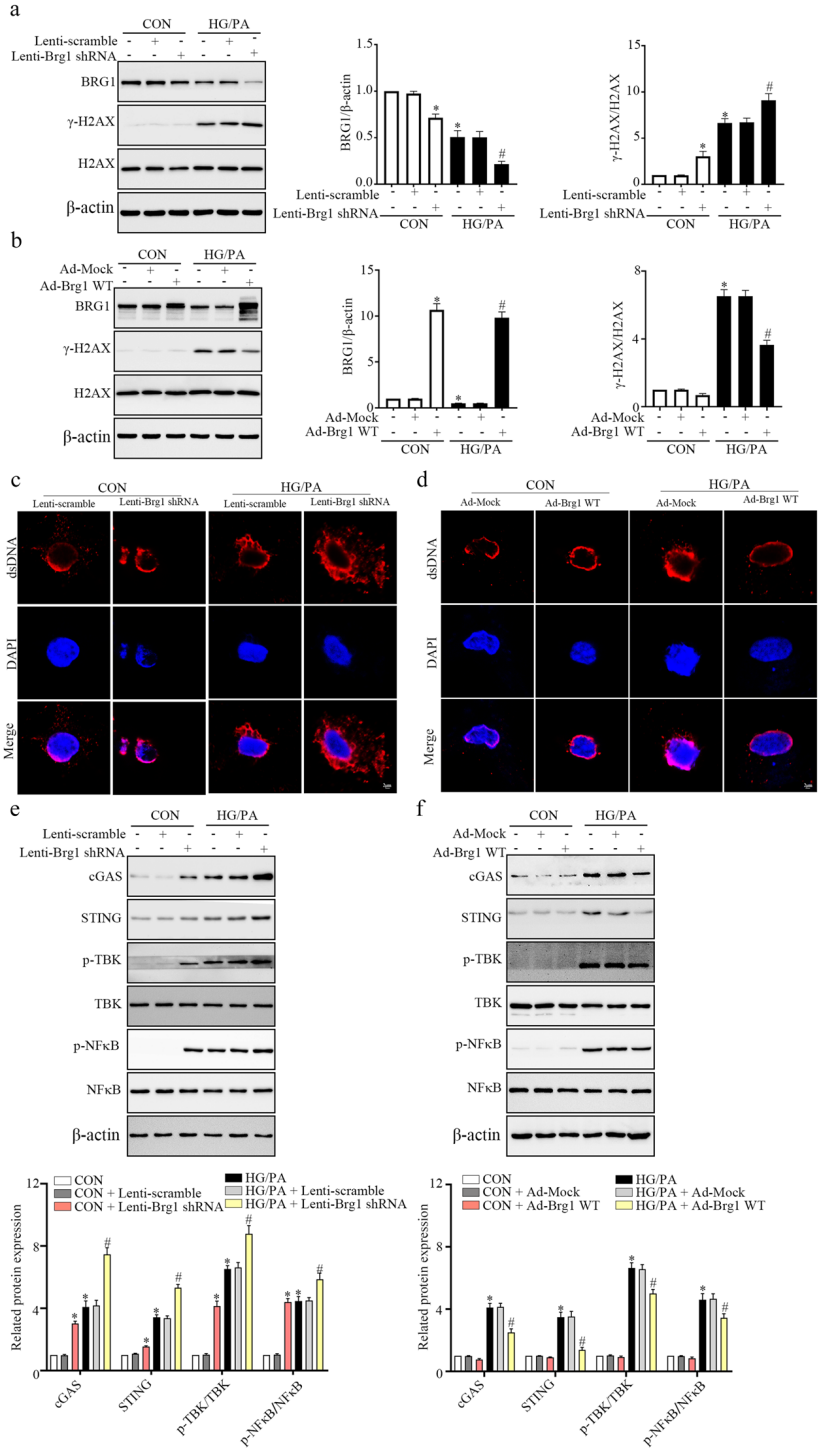
BRG1 overexpression inhibited HG/PA-induced cytoplasmic dsDNA accumulation and cGAS-STING signaling activation *in vitro*. **a**, **b** Protein levels of BRG1 and γ-H2AX in cardiomyocytes were assayed using Western blot. **c**, **d** The accumulation of dsDNA was evaluated using fluorescent staining. **e**, **f** Protein levels of cGAS-STING signaling-related genes were assayed using Western blot. * indicates *P* < 0.05 vs. the CON group, # indicates* P* < 0.05 vs. the HG/PA group.

Further, we employed NRCMs to verify the *in vivo* results. The WB results indicated that HG/PA treatment downregulated the protein level of BRG1 and upregulated the protein level of γ-H2AX in cardiomyocytes, which was consistent with the *in vivo* results (Fig. 6a and b). Moreover, HG/PA treatment increased the cytoplasmic dsDNA content in cardiomyocytes (Fig. 6c and d). We also observed that the HG/PA treatment significantly upregulated the protein levels of cGAS, STING, p-TBK and p-NF-κB in cardiomyocytes (Fig. 6e and f).

To assess the impact of BRG1 on cytoplasmic dsDNA content and activation of the cGAS-STING, we manipulated BRG1 expression in cardiomyocytes through adenovirus or lentivirus transfection for upregulation or downregulation, respectively. Knockdown of BRG1 further increased the HG/PA-induced upregulation of γ-H2AX, while BRG1 overexpression had the opposite effect (Fig. 6a and b). In addition, BRG1 knockdown enhanced HG/PA-induced cytoplasmic dsDNA accumulation, while BRG1 overexpression showed an opposite effect (Fig. 6c and d). Consistent with this, BRG1 knockdown increased the protein levels of cGAS, STING, p-TBK and p-NF-κB, whereas BRG1 overexpression had an opposite effect (Fig. 6e and f). These findings strongly suggest that BRG1 deficiency results in cytoplasmic dsDNA accumulation and cGAS-STING signaling activation.

### BRG1 Overexpression Reduction of Inflammation and Apoptosis *In Vitro*

**Fig. 7:**
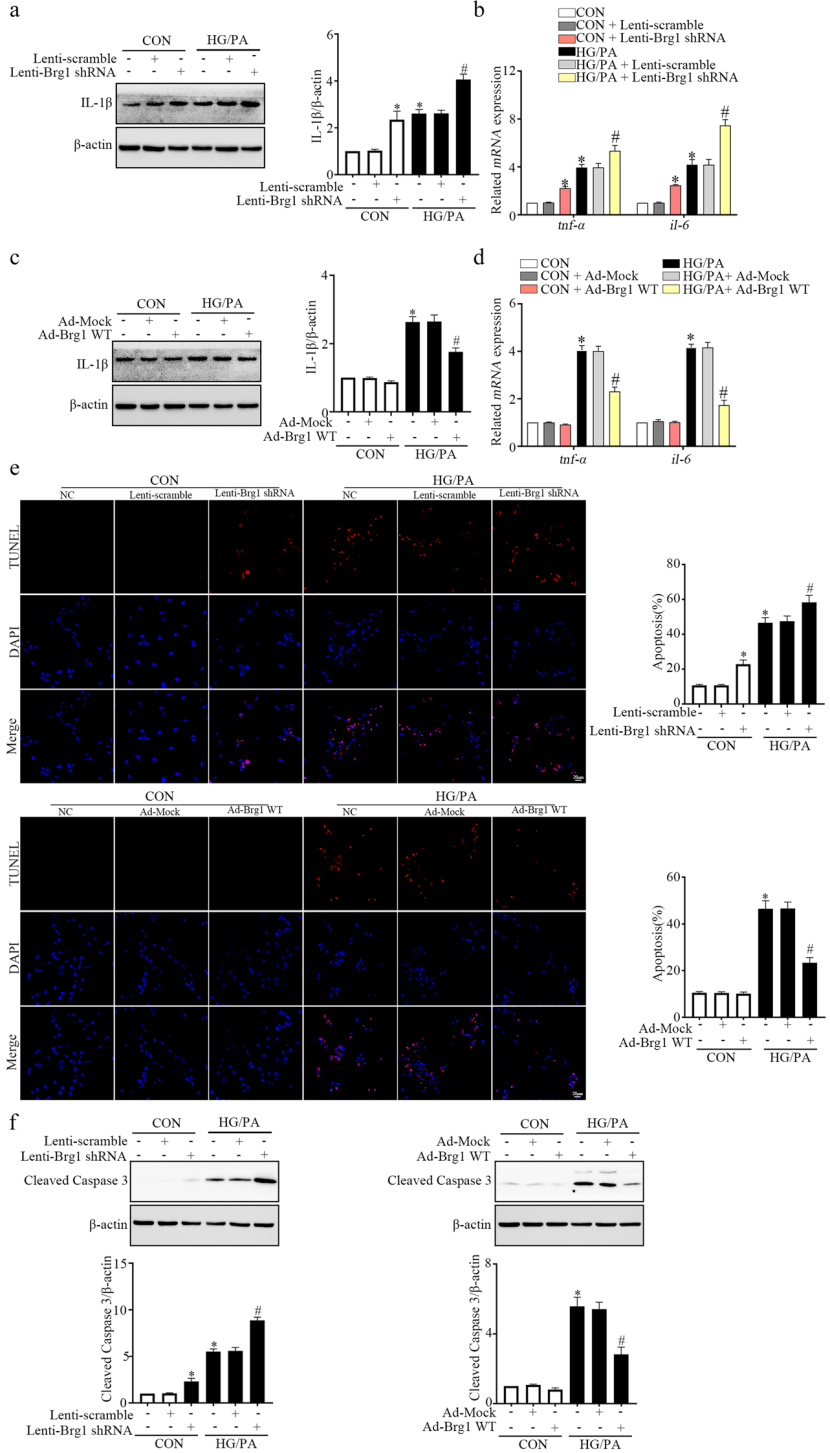
BRG1 overexpression reduction of inflammation and apoptosis *in vitro*. **a**, **c** Protein level of IL-1β was assayed using Western blot in cardiomyocytes. **b**, **d** *mRNA* levels of *tnf-α* and *il-6* were evaluated using qRT-PCR. **e** The apoptosis of cardiomyocytes was detected by the TUNEL assay. **f** Protein level of cleaved caspase-3 was assayed using Western blot. * indicates *P* < 0.05 vs. the CON group, # indicates* P* < 0.05 vs. the HG/PA group.

Next, we evaluated the function of BRG1 in HG/PA-induced cardiomyocyte inflammation and apoptosis. The WB results indicated that BRG1 knockdown further enhanced the IL-1β protein expression in HG/PA-managed cardiomyocyte, whereas BRG1 overexpressing had an opposite effect (Fig. 7a and c). The levels of *tnf-α* and *il-6* mRNA also exhibited similar trends (Fig. 7b and d). In addition, the TUNEL staining results showed that BRG1 deficiency further enhanced the apoptosis of cardiomyocytes, while BRG1 overexpression showed the opposite effect (Fig. 7e). Similarly, BRG1 knockdown upregulated the expression of cleaved caspase-3 protein, while BRG1 overexpression triggered opposite effect (Fig. 7f). These results indicated that BRG1 overexpression inhibited HG/PA-induced cardiomyocyte inflammation and apoptosis.

### Blockade of cGAS-STING Attenuated BRG1 Downregulation-induced Cardiomyocyte Inflammation and Apoptosis

To further elucidate the role of cGAS-STING in BRG1 downregulation-induced cardiomyocyte injury *in vitro*, the activity of cGAS-STING was inhibited using selective inhibitors in BRG1 knockdown and HG/PA-treated cardiomyocytes. WB analysis showed that RU.521 or C-176, a selective inhibitor for cGAS or STING, successfully inhibited cGAS or STING expression (Supplementary Fig. 2A and B).

**Fig. 8:**
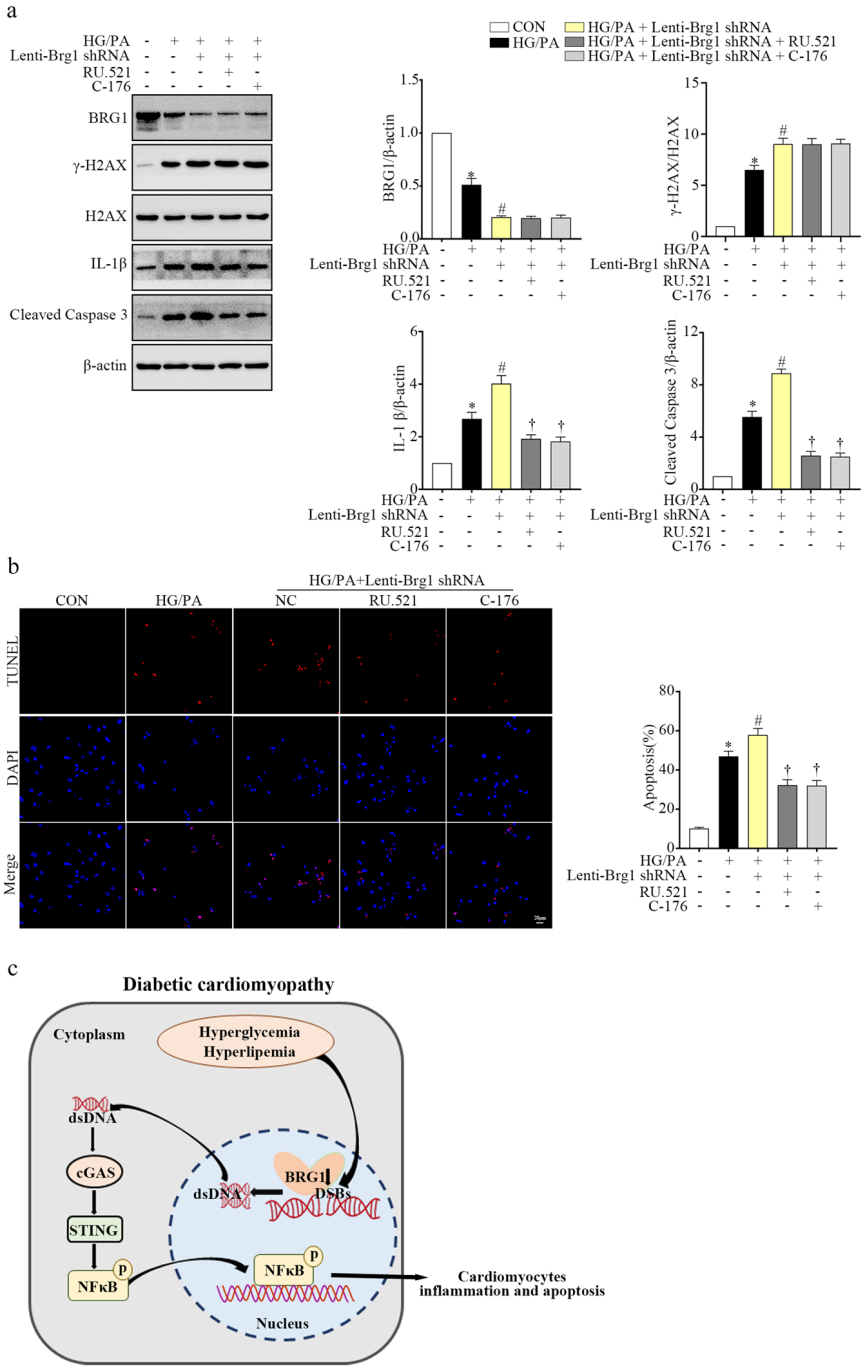
BRG1 regulates cardiomyocyte inflammation and apoptosis through cGAS-STING signaling. **a** Protein levels of BRG1, γ-H2AX, IL-1β and cleaved caspase-3 were assayed using Western blot in cardiomyocytes. **b** The apoptosis of cardiomyocytes was detected by the TUNEL assay. **c** Illustration of the proposed signaling under activation of the BRG1 deficiency mediated cGAS-STING pathway in DCM. * indicates *P* < 0.05 vs. the CON group, # indicates* P* < 0.05 vs. the HG/PA group, † indicates* P* < 0.05 vs. the HG/PA + Lenti-*Brg1* shRNA group.

Data presented in Fig. 8a indicates that both RU.521 and C-176 blocked BRG1 knockdown-induced upregulation of IL-1β and cleaved caspase-3, while RU.521 or C-176 had no effect on the expression of BRG1 and γ-H2AX (Fig. 8a). Moreover, RU.521 and C-176 inhibition alleviated apoptosis in Lenti-*Brg1* shRNA-infected NRCMs (Fig. 8b). These results suggested that BRG1 knockdown enhanced the HG/PA-induced cardiomyocyte inflammation and apoptosis *via* the cGAS-STING pathway.

Based on these results, we speculate that a hyperglycemic and hyperlipemic internal environment downregulates BRG1. This BRG1 deficiency activates the cGAS-STING by inducing dsDNA accumulation, thereby leading to cardiomyocyte inflammation and apoptosis (Fig. 8c).

## Discussion

DCM is characterized by increased cardiomyocyte apoptosis and hypertrophy, as accompanied by impaired cardiac function, which elevates the risk of HF and sudden death in DCM patients [19]. In some preliminary studies, treatments such as sodium-glucose cotransporter 2 inhibitors and dipeptidyl peptidase-4 inhibitors were found to have the potential to treat DCM [20–22]. However, their clinical efficacy is limited. Therefore, there is an urgent need to explore the mechanisms of DCM and develop effective interventions to improve the DCM symptoms. In this study, we found that BRG1 deficiency led to dsDNA accumulation, and activation of the cGAS-STING signaling in cardiomyocytes from DCM mouse model and HG/PA-cultured NRCMs. BRG1 downregulation aggravated cardiomyocyte dysfunction, resulting in cardiomyocyte inflammation and apoptosis by enhancing dsDNA accumulation and activating cGAS-STING signaling (see Fig. 8c).

BRG1 has been implicated in the pathogenesis of cardiovascular disease [23]. A previous study found that BRG1 attenuated exercise-induced physiological myocardial hypertrophy by inhibiting pressure overload-induced histone deacetylase 2 activation and serine/threonine kinase/glycogen synthase kinase 3β phosphorylation [24]. A study by Funamoto *et al*. reported that the p300/BRG1 complex promotes occurrence of heart failure by enhancing the histone globular domain H3K122 acetylation [25]. In cultured endothelial cells and arteries, proinflammatory stimuli augmented the expression of BRG1. Previous investigations have demonstrated that BRG1 contribute to inflammation and endothelial NO synthase phosphorylation thereby contributing to the development of atherosclerosis [26, 27]. Moreover, BRG1 suppressed neutrophil infiltration and modulated NO bioavailability in endothelial cells to inhibit cardiac ischemia–reperfusion injury in mice [28, 29]. In our previous study, we found that BRG1 protected the heart against acute myocardial infarction and reduced oxidative damage by activating the NRF2/HO1 signaling pathway [10]. In addition, upregulation of BRG1 expression ameliorated hyperglycemia-induced oxidative stress and cardiac hypertrophy [11, 30]. In this study, we found that BRG1 deficiency promoted the progression of DCM and aggravated cardiac dysfunction *in vivo*, demonstrating that BRG1 is a potential therapeutic target of DCM.

The pathogenesis of DCM is multifaceted, with inflammation and apoptosis emerging as significant factors. Given the limited capacity for cardiomyocyte proliferation in the adult human heart, apoptosis of cardiac muscle cells stands out as a primary contributor to cardiac remodeling and dysfunction [31, 32]. BRG1, a protein involved in various biological processes including apoptosis and inflammation, is also implicated in DCM. A recent study indicated that BRG1 overexpression attenuates apoptosis induced by high glucose exposure in retinal ganglion cells through Notch activation [33]. In addition, increased BRG1 levels have been associated with reduced inflammatory responses and decreased oxidative damage in cerebral ischemia–reperfusion injury, and BRG1 deficiency has been linked to inflammation-driven colorectal cancer [8, 34]. As a core subunit of the SWI/SNF complex, BRG1 promotes the DSBs repair by stimulating the γ-H2AX at the DSB-surrounding chromatin. Loss of BRG1 promotes DSBs repair and upregulates γ-H2AX [12, 35]. Moreover, we found that BRG1 deficiency resulted in upregulation of γ-H2AX expression *in vivo* and *in vitro*.

cGAS-STING is an evolutionarily conserved defense mechanism hat senses pathogenic DNA, and triggers the innate immune reaction by stimulating type I interferon secretion [36]. In addition, the cGAS-STING was reported to be involved in dsDNA-induced inflammation and apoptosis [37, 38]. In a previous study, doxorubicin increased the DNA damage in cardiac endothelial cells causing accumulation of dsDNA fragments and activation of cGAS-STING pathway [39]. Another recent study found that γ-H2AX upregulation may indirectly increase dsDNA accumulation in the cytoplasm [13]. In our study, we confirmed that BRG1 downregulation increased γ-H2AX expression, accompanied by the dsDNA accumulation and activation of the cGAS-STING to induce cardiomyocyte inflammation and apoptosis. A previous study indicated that failure to repair and eliminate DSBs promptly leads to the accumulation of dsDNA and subsequent apoptosis. Inhibition of cGAS has been shown to reduce cardiomyocyte apoptosis [40]. Additionally, another study demonstrated that myocardial infarction results in the release of cardiac dsDNA, and inhibiting the STING can alleviate cardiomyocyte apoptosis [41]. Our results further showed that both cGAS and STING inhibition alleviated the BRG1 knockdown-induced upregulation of IL-1β and cleaved caspase-3 in NRCMs, thereby preventing cardiomyocyte apoptosis. Together, these data demonstrate that BRG1 deficiency modulates cardiomyocyte inflammation and apoptosis by activating the cGAS-STING pathway.

Collectively, our results demonstrated that BRG1 is downregulated in hyperglycemic and hyperlipemic cardiomyocytes both *in vivo* and *in vitro*. We found that BRG1 deficiency resulted in the accumulation of dsDNA and triggered cGAS-STING activation, exacerbating cardiomyocyte inflammation and apoptosis induced by hyperglycemia and hyperlipemia. These findings suggest a potential novel therapeutic approach for managing cardiomyocyte injury in DCM.

## Supplementary Information

Below is the link to the electronic supplementary material.

Supplementary file1 (DOCX 1576 KB)

## Data Availability

No datasets were generated or analysed during the current study.
