# Supplementary material for: BRG1 Deficiency Promotes Cardiomyocyte Inflammation and Apoptosis by Activating the cGAS-STING Signaling in Diabetic Cardiomyopathy
Source: Inflammation. 2024 Jun 13;48(1):299–315. doi: 10.1007/s10753-024-02058-7 (PMC11807080; doi:10.1007/s10753-024-02058-7)
Supplement: Supplementary file 1 — Supplementary file1 (DOCX 1576 KB) [file 10753_2024_2058_MOESM1_ESM.docx]

**Supplementary**

**Table S1 The information of antibodies used in this study**

| **Name** | **Supplier** | **Cat no.** |
| --- | --- | --- |
| BRG1 | Abcam | ab110641 |
| γ-H2AX | Cell Signaling Technology | #9718 |
| H2AX | Cell Signaling Technology | #7631 |
| cGAS | Santacruz biotechnology | sc-515777 |
| STING | Cell Signaling Technology | #50494 |
| p-TBK1 | Cell Signaling Technology | #5483 |
| TBK | Cell Signaling Technology | #38066 |
| p-NFκB | Cell Signaling Technology | #3033 |
| NFκB | Cell Signaling Technology | #8242 |
| IL-1β | Abcam | ab254360 |
| Cleaved Caspase 3 | Cell Signaling Technology | #9664 |
| β-actin | Cell Signaling Technology | #8457 |
| dsDNA | Santacruz biotechnology | sc-58749 |
| α-actin | BOSTER | BM0003 |
| Dylight 549 conjugated Goat Anti-Rabbit IgG (H+L) secondary antibody | Abbkine | #A23320 |
| Dylight 549 conjugated Goat Anti-Mouse IgG (H+L) secondary antibody | Abbkine | #A23310 |
| Dylight 488 conjugated Goat Anti-Rabbit IgG (H+L) secondary antibody | Abbkine | #A23220 |
| DyLight 488 conjugated Goat Anti-Mouse IgG (H+L) secondary antibody | Abbkine | #A23210 |
| Anti-rabbit IgG (H+L), F(ab')2 Fragment (Alexa Fluor® 647 Conjugate) | Cell Signaling Technology | #4414 |

**Table S2 The primer sequence used in this study**

| **Name** | **Forward Primer** | **Reverse Primer** |
| --- | --- | --- |
| Rattus-IL-6 | GTTTCTCTCCGCAAGAGACTTC | TGTGGGTGGTATCCTCTGTGA |
| Rattus-TNF-α | GTAGCCCACGTCGTAGCAAA | AAATGGCAAATCGGCTGACG |
| Rattus-β-actin | ACCCGCGAGTACAACCTTCTT | ATATCGTCATCCATGGCGAACTGG |
| Mouse-TNF-α | CCCTCCTGGCCAACGGCATG | TCGGGGCAGCCTTGTCCCTT |
| Mouse-IL-6 | TGCCTTCTTGGGACTGATGC | GAATTGCCATTGCACAACTCT |
| Mouse-β-actin | GTGACGTTGACATCCGTAAAGA | GCCGGACTCATCGTACTCC |


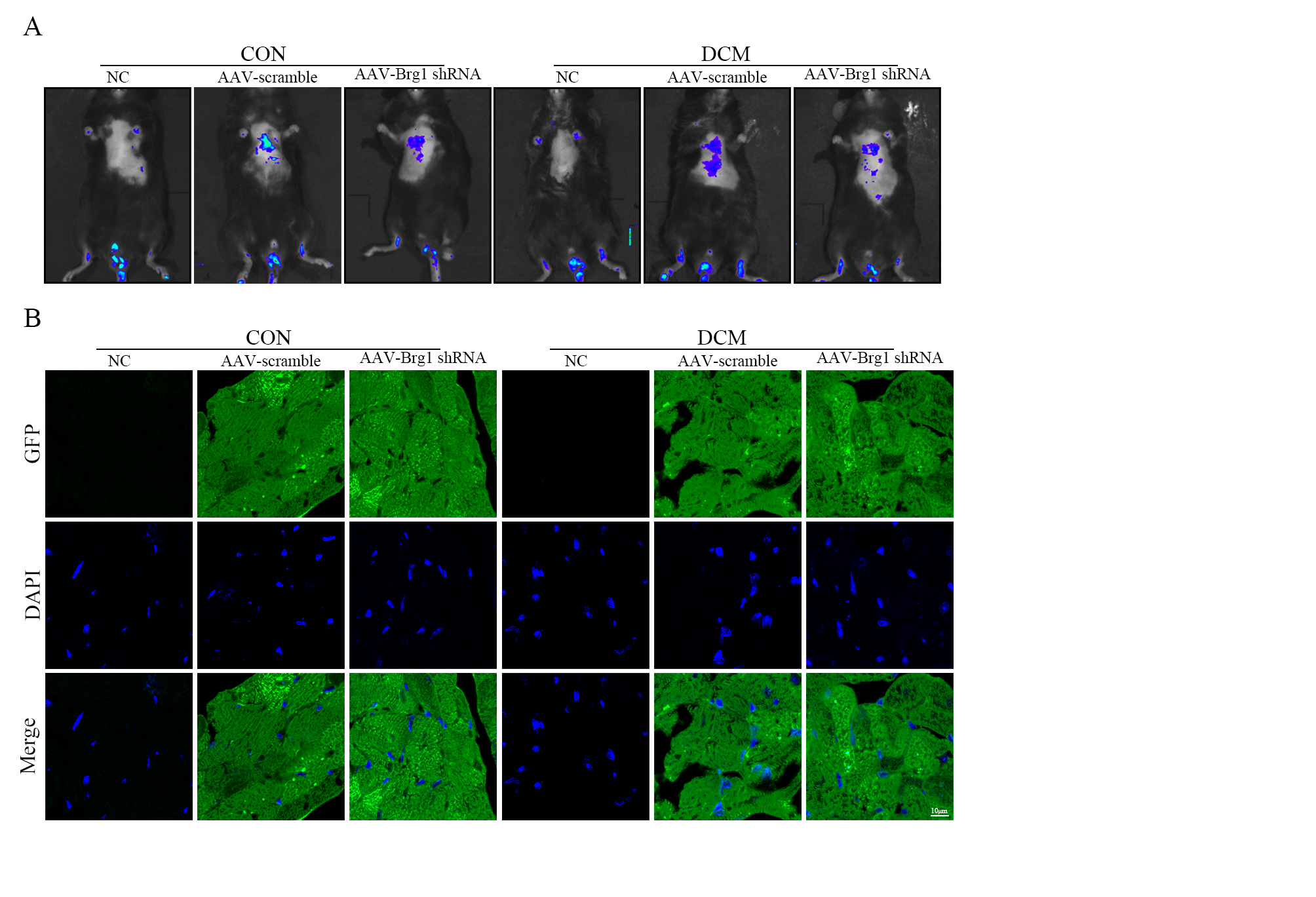


**Fig. S1 The *in vivo* imaging and GFP immunofluorescence results showed that AVV could be targeted and enriched in myocardial tissue.** (A) AAV distribution in mice was observed by *in vivo* imaging. (B) GFP immunofluorescence was used to confirm the AAV enrichment in the heart region.


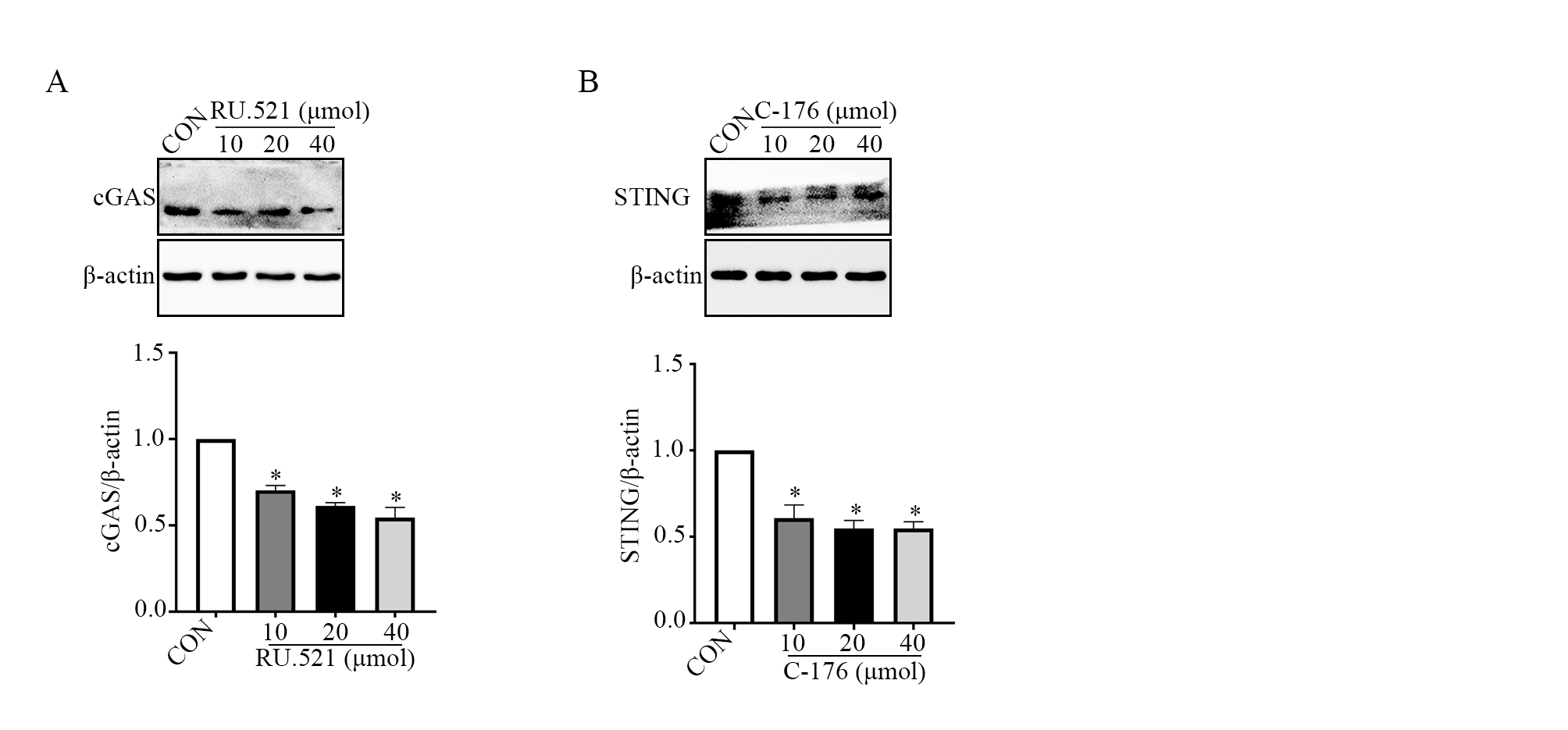


**Fig. S2 The inhibitory effect of RU.521 on cGAS (A) and C-176 on STING (B) were evaluated using Western blot.**
